# Thoughts Suggested by a Perusal of Dr. Whitmire’s Paper on Criminal Abortion, Etc.

**Published:** 1874-10

**Authors:** Geo. W. Emery


ARTICLE IV.—*Thoughts suggested by a perusal of Dr. J. S. Whitmire's Paper on Criminal Abortion, published in Journal for July*, 1874. By GEO. W. EMERY, M.D.

That the subject of criminal abortion should command the attention of profession and public, is unquestionably true. The alarming increase of the evil must be combatted, and we should endeavor to stir and agitate the public mind to not only a knowledge of the condition and state of facts, but to remedies therefor.

Dr. W. has in his article in question surveyed one of the sides of this picture to the utter neglect of the other. He commences by remarking on the general tendency to this crime, and that abortion may be produced by many different causes, telling elaborately of the *modus operandi.* The doctor, like many others who have written on this subject, informs us of the existence of law to suppress the sale of the advertised nostrums of the day, but fails to inform us of any efforts on his part to enforce the law. I would remind him of the axiom that "example is better than precept." Have we not, as a profession, enough moralizing on the nature and diagnosis of this *society criminal plague*, and has not the *modus operandi* of this crime, so often detailed by the secular and religious press, become a matter of household knowledge, until we may almost fear that it is not improper to modify Pope, (as far as this subject is concerned,)

'That seen so oft, familiar with his face,
We now endure, and pity, and embrace."

I hope we may in the future have more thought directed to the treatment of this crime, and with this aim in view, would now suggest some thoughts tending in this direction, and divide these into—

1st. The reason for the failure of the laws already enacted.

2nd. Methods which, in the writer's judgment, may assist in decreasing or limiting the evil.

One principal cause for want of enforcement of the law, in my judgment, is found in the fact that an attempt usually involves so great a loss of time and expense, with (if proven) such doubtful results as to just punishment. (See the action of the jury in recent case of Earll, the murderer, for an utter failure of an adequate punishment for the crime committed.) Can any honest, candid mind read with composure the fact printed and sent broadcast throughout the land, that a part of that jury argued the want of crime in the act of that *lecherous thug*. If those men spoke the honest conviction of their hearts, and I have heard many expressions earnestly and sincerely made of a similar nature, then we should first educate the people as a mass to the science of the nineteenth century, in the fact that abortions at any period after the discovery of pregnancy (except in necessary cases, and then not till after medical counsel,) is murder.

I care not what the common law calls it, that is what must be applied to this act by candid investigators of the subject. As another reason for this failure, I believe the aiders and abettors in this class of crime are not properly punished. Where is he who, after causing the trouble, assisted in and winked at the murder of Rosetta Jackson? Also those persons in whose house the unfortunate but principal actor offered up her life as the sacrifice? I say, where are they? and how much less criminal is that barber than was Earll? With what zeal did they who induced with lucre this vampire to the crime, attempt to smother the villainy, and how easily were they permitted to return to the comforts of social life. May we not ask, is this justice and equity? Compare it with other classes of criminal transgressions; are not the accomplices held guilty and punished with the perpetrators? This leads me to the second division of my subject, viz., the consideration of methods to prevent this crime. I feel that this will not be accomplished, while this crime is recorded on our statute books by the gentle appellation of a high misdemeanor. Let us, as a profession, urge an alteration in this term, and have the act classed where it belongs, viz., with capital crime, and the penalty thereto attached.

In order to arrest the sale of the nostrums whose sole object is to assist in this nefarious work, I would suggest that the wholesale drug houses be subjected to inspection, and all remedies of this class confiscated, and that the expense for the time and trouble, with costs of suit, be also assessed against any house guilty of such sale. This would effectually cut off the supply of this class of remedies from the country drug stores.

In conclusion, I would suggest that the aiders and abettors in this crime should be subjected to severe penalty, and thus taught that they cannot induce a crime, and having by perjury and other means attempted to cover it, walk off rubbing their hands in glee, to think that they may always have a scapegoat and victim for a sacrifice in the medical scoundrel who thus debases his profession.

